# GPTree Cluster: phylogenetic tree cluster generator in the context of supertree inference

**DOI:** 10.1093/bioadv/vbad023

**Published:** 2023-03-03

**Authors:** Aleksandr Koshkarov, Nadia Tahiri

**Affiliations:** Department of Computer Science, University of Sherbrooke, 2500, Boulevard de l’Université, Sherbrooke, Québec J1K 2R1, Canada; Department of Computer Science, University of Sherbrooke, 2500, Boulevard de l’Université, Sherbrooke, Québec J1K 2R1, Canadanadia.tahiri@usherbrooke.ca

## Abstract

**Summary:**

For many years, evolutionary and molecular biologists have been working with phylogenetic supertrees, which are oriented acyclic graph structures. In the standard approaches, supertrees are obtained by concatenating a set of phylogenetic trees defined on different but overlapping sets of taxa (i.e. species). More recent approaches propose alternative solutions for supertree inference. The testing of new metrics for comparing supertrees and adapting clustering algorithms to overlapping phylogenetic trees with different numbers of leaves requires large amounts of data. In this context, designing a new approach and developing a computer program to generate phylogenetic tree clusters with different numbers of overlapping leaves are key elements to advance research on phylogenetic supertrees and evolution. The main objective of the project is to propose a new approach to simulate clusters of phylogenetic trees defined on different, but mutually overlapping, sets of taxa, with biological events. The proposed generator can be used to generate a certain number of clusters of phylogenetic trees in Newick format with a variable number of leaves and with a defined level of overlap between trees in clusters.

**Availability and implementation:**

A Python script version 3.7, called GPTree Cluster, which implements the discussed approach, is freely available at: https://github.com/tahiri-lab/GPTree/tree/GPTreeCluster

## 1 Introduction

For many years, evolutionary and molecular biologists have been working with phylogenetic trees, which are oriented acyclic graph structures corresponding to schematic representations of the evolutionary history of species. In addition, biologists often deal with supertrees, which consist of a combination of a set of phylogenetic trees defined on different, but mutually overlapping, sets of taxa. The study of these structures is essential for many evolutionary researches, including the Open Tree Online Project (OpenTree) (Hinchliff *et al.*, 2015) corresponding to the new version of Tree of Life (ToL) (Steel and Bocker, 2000), which contributes to a better understanding of species biodiversity.

In these specific circumstances, the process of obtaining a supertree can be divided into two steps (Tahiri *et al.*, 2022a). The first step in the process is the inference of small trees defined on different, but mutually overlapping taxa. The second step consists of the combination of these trees into one or more supertrees. The solution returning a unique supertree (i.e. homogeneous data set) is obtained by using existing supertree reconstruction methods, e.g. Matrix Representation with Parsimony (Bininda-Emonds and Sanderson, 2001), which allow combining trees defined on different taxon sets that perform the matrix-like aggregation of the given partial trees. The solution that returns an alternative supertree (i.e. heterogeneous data set) is produced by clustering a set of phylogenetic trees (e.g. Guénoche, 2013; Tahiri *et al.*, 2018, 2022a). The alternative approach is much more robust, and more accurate by preserving the diversity of the input phylogenetic trees through the often best-resolved supertrees.

Clustering plays a special role in detecting biodiversity, which can be applied to a set of trees for subsequent supertree inference from them. Given clusters of phylogenetic trees with the above characteristics, the process of inferring supertrees using existing approaches becomes easier and more accessible to researchers for their relevant tasks (e.g. Swenson *et al.*, 2010; Tahiri *et al.*, 2022a).

For scientific experiments, this kind of clusters can be obtained by clustering a large set of data or by generating predefined clusters of phylogenetic trees for further study. In this case, we consider simulated data for use in testing various hypotheses.

Therefore, the problem of obtaining phylogenetic tree data with different numbers of overlapping leaves for the testing and adaptation tasks of clustering and supertree experiment algorithms is ongoing. At the same time, the generated data should also reflect some biological events, such as horizontal gene transfers (HGTs) or hybridization, which plays a crucial role in evolutionary processes (Wolfe and Fournier, 2018).

In this article, we describe a new approach to generate clusters of phylogenetic trees with a different number of leaves and a defined level of overlap. The article is organized into four sections. In the first section, we present a brief literature review of existing phylogenetic tree generators. In the second section, we describe the workflow of the proposed tree generator with implemented HGT. In the third section, we show an example of a cluster of generated trees and provide an analysis of HGT. In the last section, we discuss use cases and possible extensions of the proposed approach.

## 2 Related work

The task of data generation is frequently encountered in the field of bioinformatics. However, analysis of the literature has not revealed data generators capable of simulating clusters of phylogenetic trees with a variable number of leaves and with the condition of overlap between trees. At the same time, there are several different phylogenetic tree generators, which, potentially, can be used as a basis for other, more advanced implementations.

Existing simulators can be divided into several categories depending on the programming language used (i.e. C, Java, Python) and the internal properties of the generated trees. For example, there are generators of random phylogenetic trees with customizable parameters such as the number of trees generated, the number of leaves in each tree and the average branch length. These tools include T-Rex (Tree and Reticulogram Reconstruction) (Boc *et al.*, 2012; Makarenkov, 2001), made in C++, with the module ‘*Random tree generator*’, and the library ete3 (Huerta-Cepas *et al.*, 2016) with the function *populate()*, developed in Python. Horiike *et al.* (2011) proposed a generator, made in Perl that adds HGT to trees.

More advanced phylogenetic tree simulators include the incorporation of additional features. Mallo *et al.* (2016) developed in C the SimPhy tool with the following features: lineage sorting, gene duplication, gene loss and HGT. Packages developed in Java include HybridSym, SaGePy and GenPhyloData. HybridSym (Woodhams *et al.*, 2016) provides features for divergence speciation, hybrid speciation, and introgression. SaGePy (Kundu and Bansal, 2019) has functionality for using and configuring generation parameters such as gene birth, speciation, gene duplication, HGT and gene losses. GenPhyloData (Sjöstrand *et al.*, 2013) includes gene duplication, gene loss, lateral gene transfer, clock models and sequence evolution.

In addition, there are solutions in R, such as the Castor package (Louca and Doebeli, 2018), which simulates events including HGTs, gene duplication and gene loss for the generation of phylogenetic trees. Finally, the Python tools should also be considered. We have already mentioned above the library ete3 for random tree generation. More functional Python packages include Zombi (with birth-death model) (Davín *et al.*, 2020), AsymmeTree (with speciation, duplication, loss, gene conversion and HGT events) (Schaller *et al.*, 2022), and Ngesh (with mutation events, birth and death rates and HGT) (Tresoldi, 2021).

In this article, the main language used for prototyping and testing the solution is Python, which has a vast arsenal of libraries for processing data of different structures, visualizing results and using the capabilities of machine learning models (e.g. classification and clustering tasks). From this perspective, we consider the use of a basic generator in Python with the ability to incorporate biological events (e.g. HGTs) into the generated phylogenetic trees. These characteristics apply to the library AsymmeTree, whose authors in their article (Schaller *et al.*, 2022) showed its advantage over other similar solutions in Python.

## 3 Methods

In order to generate clusters of phylogenetic trees in the context of supertree inference, the following requirements should be satisfied for the generator:

1. The number of clusters of phylogenetic trees (gene trees) to be generated is required;
2. Trees should have a different number of leaves within a specified range;
3. Trees in a cluster should overlap at a certain level;
4. There should be a specific number of trees in each cluster;
5. Trees in each cluster should include biological events (i.e. HGT).

**Fig. 1.**
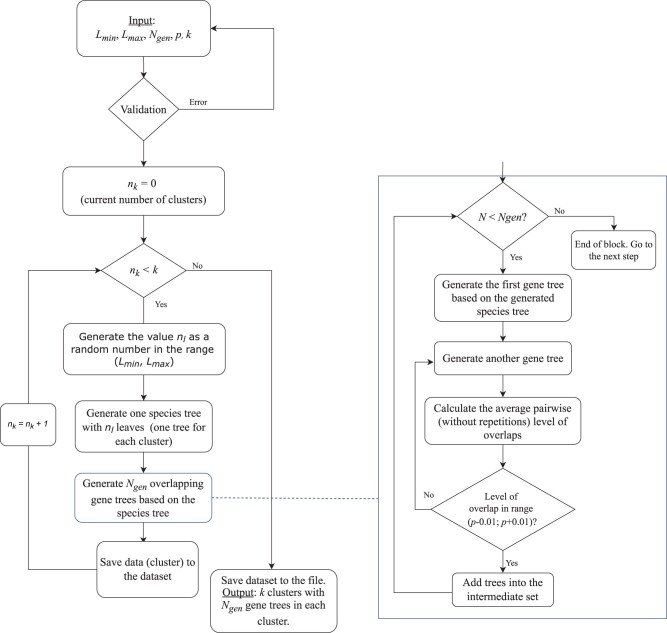
The scheme of generation of phylogenetic tree clusters. The procedure consists of three main blocks. In the first block, the user has to set the initial parameters, including the number of clusters, the minimum and maximum possible number of leaves for trees in a cluster, the number of trees to be generated for each cluster and the average level of overlap (common leaves) between trees in a cluster. The second block includes the generation of a species tree (one tree for each cluster), and a specified number of gene trees based on it with the implementation of horizontal gene transfer (HGT). The third block is responsible for selecting overlapping trees with a given level of overlap to add them to the cluster. As a result, the user obtains clusters of phylogenetic trees with specified parameters

An analysis of existing phylogenetic and bioinformatics tools did not identify the availability of phylogenetic tree cluster generators satisfying the mentioned conditions. In this context, we propose a generator to address this limitation. A graphical representation of the pipeline is presented in Figure 1. The principal steps of the proposed workflow are as follows:

1. The algorithm considers five input parameters described as follows:
  - *k* is the number of clusters of phylogenetic trees to be generated;
  - *L*_min_ is the minimum limit by the number of leaves in the generated phylogenetic trees;
  - *L*_max_ is the maximum limit by the number of leaves in the generated phylogenetic trees;
  - *N*_gen_ is the number of phylogenetic trees in each cluster;
  - *p* is the level (in decimal) of overlap between phylogenetic trees in a cluster.
2. The generation of a species tree with a random number of leaves in the interval [*L*_min_, *L*_max_]. This tree is the base tree for the generation of gene trees within each cluster and it is considered as a centroid of the cluster. The species tree is simulated using the Yule model (Schaller *et al.*, 2022), and, in fact, the tree is planted root, i.e. the root has a single descendant.
3. The generation of *N*_gen_ overlapping gene trees based on the species tree generated in the previous step performs in four substeps which are described as follows:
  - Generation of the first gene tree based on the generated species tree. Gene trees are modeled using the birth-death process (Schaller *et al.*, 2022) with the implementation of gene duplications, losses, HGT and gene conversion which are customizable parameters.
  - Generation of the next gene tree.
  - Examination of the level of overlapping between the existing gene trees. If the condition is satisfied [i.e. the level of overlap in the range (*p* − 0.01; *p* + 0.01)], add the current gene tree to the cluster. Repeat until the specified number (*N*_gen_) of trees in the cluster is reached.
4. Generation of trees for the next cluster for cases *k* > 1.
5. Preservation the generated dataset of clusters of phylogenetic trees (in the Newick format) into a text file.

All of these steps are shown in detail in Figure 1. The workflow consists of three main blocks. The first block consists of the validation of a set of parameters essential for the generation of phylogenetic tree clusters, e.g. the number of clusters, the minimum and maximum possible number of leaves, the number of trees to be generated for each cluster and the average level of overlap (common leaves) between the trees in a cluster. The second block includes the generation of a species tree (one tree for each cluster), and a specified number of gene trees based on it with the implementation of HGT. The third block is responsible for selecting overlapping trees. In our current implementation, the duplication rate is set to 0 by default, therefore the results do not produce multi-labelled trees.

The level of overlap between two trees we determine by the following Equation (1) (also known as the Jaccard Similarity Coefficient): where $OL(T_{1},T_{2})$ is the overlap level between tree 1 (denoted *T*_1_) and tree 2 (denoted $T_{2}),\;n(T_{1},T_{2})$ is the number of common leaves between *T*_1_ and *T*_2_, $n(T_{1})$ is the number of leaves in *T*_1_, $n(T_{2})$ is the number of leaves in *T*_2_.


(1) $$OL(T_{1},T_{2})=\frac{n(T_{1},T_{2})}{n(T_{1})+n(T_{2})-n(T_{1},T_{2})},$$

We can alternatively use the next equation for the overlap coefficient (*OC*), which is defined, in this case, as the number of leaves common to tree *T*_1_ and tree *T*_2_ over the minimum number of leaves between tree *T*_1_ and *T*_2_: where $OC(T_{1},T_{2})$ is the overlap level between *T*_1_ and *T*_2_.


(2) $$OC(T_{1},T_{2})=\frac{n(T_{1},T_{2})}{\min(n(T_{1}),n(T_{2}))},$$

**Fig. 2.**
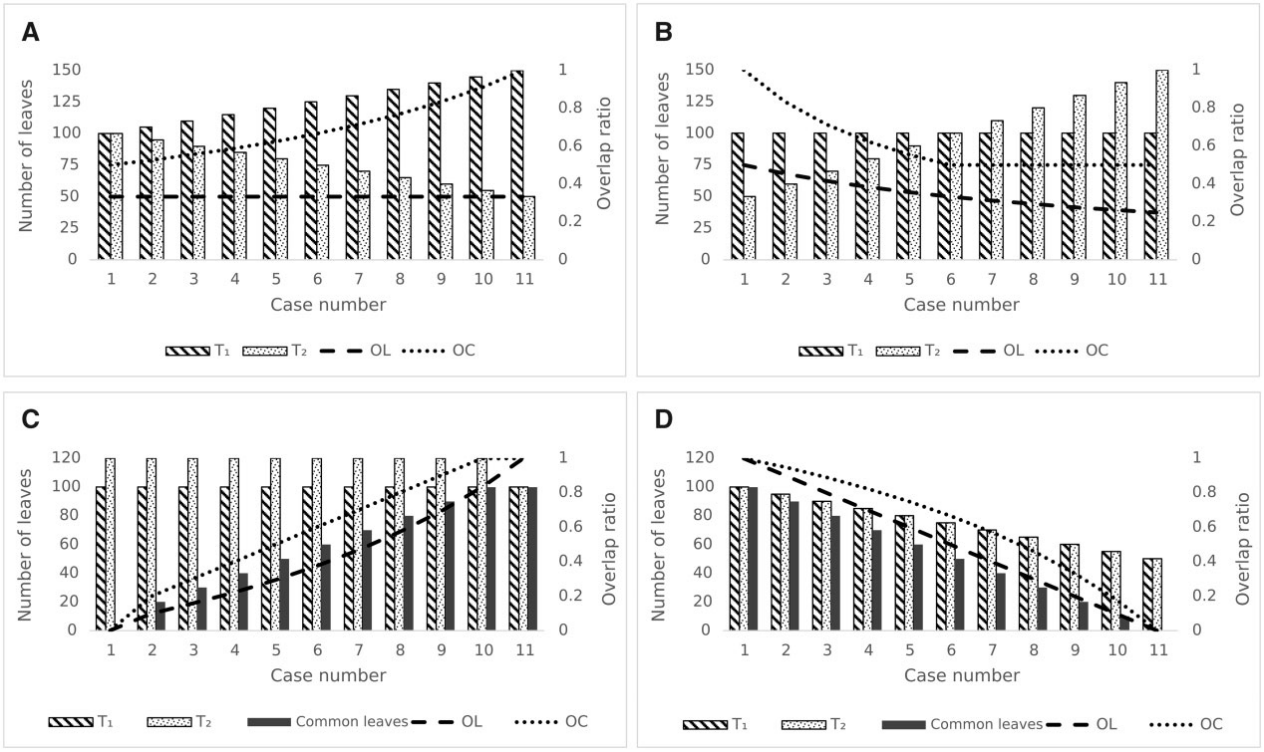
Comparison of species set overlap between phylogenetic trees *T*_1_ and *T*_2_ in 11 study cases by varying the number of species. (**A**) The visualization when the number of common leaves is constant (in particular, 50) and the sum of leaves of both trees is constant (one tree increases the number of leaves, and the second tree decreases it by the same value) is shown. *OL* has a permanent value under these conditions. (**B**) It deals with a scenario in which the number of leaves of one tree (*T*_1_) is constant (100), the number of common leaves is constant (50) and the number of leaves of the second tree (*T*_2_) is variable (in the beginning it is less than the first tree and then it is greater). (**C**) Situations where the number of leaves of both trees remains unchanged, but the number of common leaves changes (increases) is shown. (**D**) It considers a scenario in which both trees have the same number of leaves and the number of common leaves varies. The left vertical axis shows the number of leaves, the right vertical axis shows *OL* and *OC* values, and the bottom horizontal axis is used for the number of tested cases

We analyzed four basic scenarios (denoted A, B, C and D in Fig. 2) depending on the level of overlap between species sets in different phylogenetic trees (with 11 cases of the number of leaves in both trees and the number of common leaves for each scenario, see Fig. 2):

1. Constant number of common leaves in the trees (in this scenario, this value is equal to 50) and a variable number of leaves in the two compared trees, but the sum of the number of these leaves is constant (Fig. 2A). This scenario shows a situation, where the Overlap Level (*OL*) remains constant.
2. Constant number of common leaves in the trees (in this scenario this value is 50) and a varying number of leaves in one of the trees (in the other tree this value is constant) (Fig. 2B). The situation when the number of leaves of each tree becomes equal and then the number of leaves of the second tree increases is remarkable. In this case, *OC* takes a constant value (it can be estimated as a fraction of the number of total leaves relative to the number of leaves of the smaller tree). This is a particular case, which can have different variations [a consequence of Equation (2)].
3. Constant number of leaves in both trees, but the variable value of common leaves between these trees (Figure 2C). We can see that these values can be the same at the extremes (i.e. when *OL* and *OC* are 0 and 1).
4. Both trees have the same number of leaves. The number of common leaves varies. Figure 2D shows a decrease in the number of leaves in the trees and a decrease in the number of common leaves (from case 1 to 11).

These four scenarios show the behavior of the two overlap indicators depending on the size of trees (number of leaves) and the number of common leaves. If we compare the values of $OL(T_{1},T_{2})$ and $OC(T_{1},T_{2})$, we can see that $OC(T_{1},T_{2})$ will be larger than $OL(T_{1},T_{2})$ due to the smaller value in the denominator (except for cases when they are equal as shown in Scenario 3). For the case of fully overlapping trees, both values are equal to 1, and for the case of non-overlapping trees ($n(T_{1},T_{2})=0$), these coefficients are equal to 0. Based on our experiments, we chose Equation (1) as the basic option, but our software solution offers the choice to select either Equation (1) or (2) to calculate the level of overlap between trees.

## 4 Results and discussion

The proposed workflow is the basis of the solution developed in Python 3.7. This software is freely available on the GitHub platform (https://github.com/tahiri-lab/GPTree/tree/GPTreeCluster).

In this work, we used the AsymmeTree version 2.2 library as a core component, which simulates species trees and gene trees with HGT. This library has the essential features we specified for the single tree generation block including evolutionary scenarios involving duplication, loss, and HGT events. In addition, the library is written in Python which made its incorporation into our supertree generation tool more intuitive and made it possible to configure additional parameters such as birth rate, loss rate and noise.

**Fig. 3.**
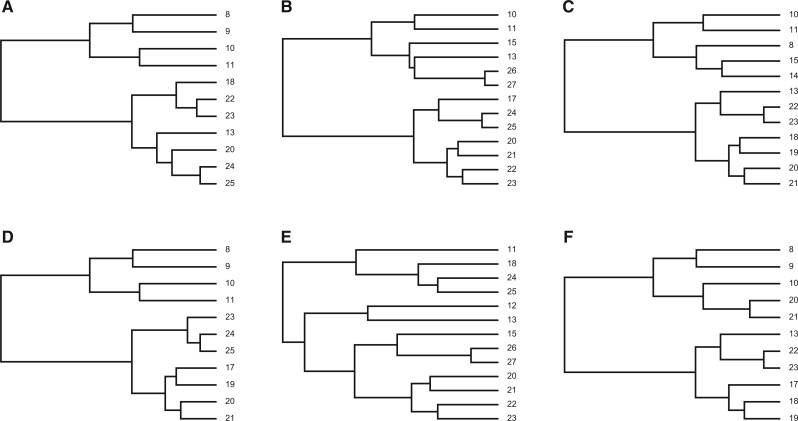
Visualization of a cluster of six trees from the generated dataset. The initial parameters used are the number of clusters *k *=* *3 (but only the first cluster is visualized), the minimum number of leaves *L*_min_ = 10, the maximum number of leaves *L*_max_ = 15, the number of phylogenetic trees for a cluster *N*_gen_ = 6 and the level of overlap *p *=* *0.5. (**A**) Representing phylogenetic tree 1 (denoted *T*_1_), (**B**) representing phylogenetic tree 2 (denoted *T*_2_), (**C**) Representing phylogenetic tree 1 (denoted *T*_3_), (**D**) representing phylogenetic tree 4 (denoted *T*_4_), (**E**) representing phylogenetic tree 5 (denoted *T*_5_) and (**F**) representing phylogenetic tree 6 (denoted *T*_6_)

We tested the software solution in the case of generating multiple phylogenetic tree clusters with overlap. An example of cluster generation of six trees with the number of leaves in the range [10, 15] and the overlap level of 0.5 is shown in Figure 3. In the visualization, we can see the variation in the number of leaves in a given range and the level of common leaves in the tree cluster. In order to examine it in detail, we provide additional calculations in the Table 1 with the number of common leaves and overlap levels for all six trees in this cluster. The calculations show that the average level of overlap is 0.505. These values correspond to the conditions of data generation.

**Table 1.** Calculation of common leaves and overlap level (*OL*) between trees in the cluster

Trees	Leaves	*OL* (overlap level)/common leaves					
		*T*_1_	*T*_2_	*T*_3_	*T*_4_	*T*_5_	*T*_6_
*T*_1_	11		8	8	8	8	8
*T*_2_	13	0.5		8	8	11	7
*T*_3_	12	0.533	0.471		7	8	9
*T*_4_	11	0.571	0.5	0.438		6	8
*T*_5_	13	0.5	0.733	0.471	0.333		6
*T*_6_	11	0.571	0.412	0.643	0.571	0.333	

The table shows the number of leaves in each tree of the indicated cluster, the number of common leaves in each pair of six trees (upper right part of the table), and the pairwise calculation of overlap level (*OL*) according to Equation (1) (bottom left part of the table).

**Fig. 4.**
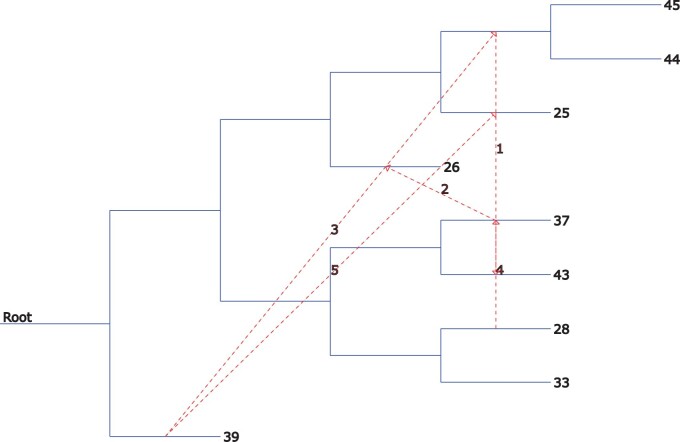
Visualization of the HGT analysis in the generated data using one pair of species tree and gene tree as an example. Five unique scenarios of HGT have been identified. HGT 1 corresponds to the transfer from specie 39 to subtree (43, 44, 45); HGT 2 is the transfer from specie 37 to specie 26; HGT 3 is the transfer from specie 39 to subtree (43, 44, 45); HGT 4 is the transfer from specie 28 to subtree (26, 37); and HGT 5 is the transfer from subtree (39, 43, 44, 45) to specie 25. The analysis was performed using the T-Rex web service

In addition, during the testing process, a pair of species trees and gene trees were saved for analysis on the HGT. This analysis was performed using the T-Rex (Boc *et al.*, 2010) web service by iterating multiple HGTs and bipartition dissimilarity. The results and details are presented in Figure 4.

Using the basic properties of the generated dataset, including the level of overlap, different numbers of leaves, and biological events, it is possible to obtain a dataset of phylogenetic tree clusters and then infer supertrees for each cluster. In this context, the workflow can be extended with an additional block responsible for supertrees, where a supertree will be inferred for each cluster. For example, the CLANN (Creevey and McInerney, 2005) tool can be used for this purpose, and its documentation contains complete instructions.

The following scenarios of usage of the developed tool can be highlighted:

1. Generation of clusters of phylogenetic trees for inference supertrees. If we extend the solution as described above, we can get a supertree generator, where *k* is the number of generated supertrees (one supertree per cluster). This can be further used for supertree comparison tasks (in terms of new metrics).
2. Utilization of the generator and the output dataset for adapting and testing clustering algorithms for trees with different numbers of leaves and overlaps.
3. Generation of tree clusters to create a training (labeled) dataset for a machine learning model (e.g. convolutional neural network or transformer) for the task of classifying phylogenetic tree data. In particular, such a trained model can be used to estimate (predict) the number of clusters in the studied data to further customize the phylogenetic tree clustering algorithm [see example in Tahiri *et al.* (2022b)].

The solution is developed in two formats: Jupiter notebook and *.py module. The first version can be run in Google Colaboratory for experiments with data generation, data visualization, and analysis of additional parameters, if necessary according to the objectives. The second version can be used standalone or as part of another tool, where the corresponding data generation is needed. The module version requires passing several initial parameters [number of clusters (*k*), minimum possible number of leaves for trees in a cluster (*L*_min_), maximum possible number of leaves for trees in a cluster (*L*_max_), number of trees to be generated in each cluster (*N*_gen_) and average level of overlap (common leaves) between trees in each cluster (*p*)]. As a result, a generated dataset of the specified number of trees (separated by clusters) is saved in Newick format. All these files are open source and are stored in the project repository on GitHub.

It is worth noting potential additional steps to improve the solution. In particular, the program can be considered in a modular format, where it is possible to change the basic generator, add distance metrics (within each cluster and between clusters), and provide unique leaf names for trees of different clusters if it corresponds to the objectives. Moreover, this format has the capability to incorporate additional tools, such as a coalescence simulation tool, especially in cases where the use of gene trees is aimed at incomplete lineage sorting.

In our solution, we define an average level of overlap. This level has left and right limits, but the user can extend these limits (while keeping the average level unchanged) if necessary for their objectives. On the basis of this, we are going to use the overlap limits (the left and right limits) as additional parameters that can be set by the user in the future version of our tool.

In addition, it is possible to estimate the computational complexity of the proposed solution. The complexity is linear O(N), where *N* is the total number of trees the user needs to generate. Furthermore, this value is the sum of the number of phylogenetic trees per cluster. The complexity also depends on the number of leaves *n*. Finally, the complexity increases to a quadratic value $O(nN^{2})$ due to the calculation of the number of common leaves.

## 5 Conclusion

In this article, an approach to generating clusters of phylogenetic trees with overlaps and different numbers of leaves was proposed. Based on this, a software solution was developed in Python version 3.7, freely available to the scientific community on GitHub. The simulated data (saved in Newick format) contains biological events (in particular, HGT) that bring the generated data closer to the real data. The current solution is presented as a Jupyter Notebook and as a Python module that can be used as part of another tool that requires relevant data. These formats make the solution easy for potential users to utilize.

The solution will be useful for researchers in phylogeny, bioinformatics, biology, and other related fields for the tasks of phylogenetic tree clustering (testing algorithms and metrics), and working with phylogenetic supertrees. The tool can be extended by connecting new modules—for example, for supertree inference. Moreover, additional parameter customization (and flexibility in terms of the use of its individual components) can expand the options for using the solution.
